# A comparative overview of aggregation methods of a graphical risk assessment: An analysis based on a critical infrastructure project

**DOI:** 10.1371/journal.pone.0325267

**Published:** 2025-06-05

**Authors:** Albert Kutej, Stefan Rass, Rainer W. Alexandrowicz

**Affiliations:** 1 University of Klagenfurt, Klagenfurt, Austria; 2 Secure Systems Group, LIT Secure and Correct Systems Lab, Johannes Kepler University, Linz, Austria; 3 Department of Methodology, Institute of Psychology, University of Klagenfurt, Klagenfurt, Austria; Indian Institute of Technology Madras, INDIA

## Abstract

We compare the traditional risk and opportunity assessment method, which relies on fixed values for impacts (or potentials) and probabilities, with a graphical approach that incorporates the representation of uncertainties. To date, this graphical risk assessment method, in combination with subsequent opinion pooling, has neither been empirically studied nor validated. Therefore, its comparison with classical risk assessment remains an open question. Its significance lies in the need to validate the graphical method as a consistent generalization of the well-established classical risk assessment, which is based on expert-defined impact and probability specifications. To establish and test consistency between classical and graphical risk specifications, the latter requires appropriate aggregation methods to synthesize risk assessments from individual expert judgments—an independent challenge in itself. Therefore, various aggregation methods are introduced and tested using a case study in the field of critical infrastructure, based on expert interviews from multiple specialized domains. The collected data enables both qualitative and statistical analysis. The Kolmogorov-Smirnov and Wasserstein tests were employed to quantify differences between the methods, while overall significance was assessed using Fisher’s method. This study underscores the importance of integrating uncertainties into risk assessments and provides insights into the effectiveness and applicability of different aggregation methods.

## 1. Introduction

Critical infrastructures are subject to particularly stringent risk management requirements (e.g., the recent NIS2 directive [1] and the EU’s Cyber Resilience Act [2]), while established standards such as ISO 31000 [3] provide well-proven methods for assessing likelihoods and impacts. However, working with overly precise values for the impacts and likelihoods of security threats has been discouraged [4], leading to a generalization of risk assessment methods that allow specifying risk-related data along with associated uncertainties [5]. Nevertheless, a critical infrastructure provider will inevitably need to consult multiple experts to obtain a reasonably accurate risk picture. While subjective risk specifications in quantitative or categorical risk assessment can be aggregated in various ways [6], generalized risk assessment methods do not have direct counterparts for the same problem. This motivates an investigation into aggregation methods for “generalized” risk specification techniques, such as the graphical tool proposed in [5]. However, as with any generalization, the outcomes should remain consistent with those obtained from classical risk assessment and aggregation methods. To this end, we conducted a study in a real-world critical infrastructure setting to compare the results of graphical and classical risk assessments under different aggregation approaches. Consider that you are the CEO of a hospital and must decide whether the occurrence of a power blackout is (a) likely to happen and (b) will cause severe harm to your company. Furthermore, imagine that you must make such decisions regarding several potential threats, ensuring that your resource allocation is appropriate to defend against these known risks. Risk matrices are a traditional tool to (visually) organize risks in terms of impact (*losses*) and likelihood for a respective ranking about which risks or threats to address first (see Fig 2 for an example).

**Fig 1:**
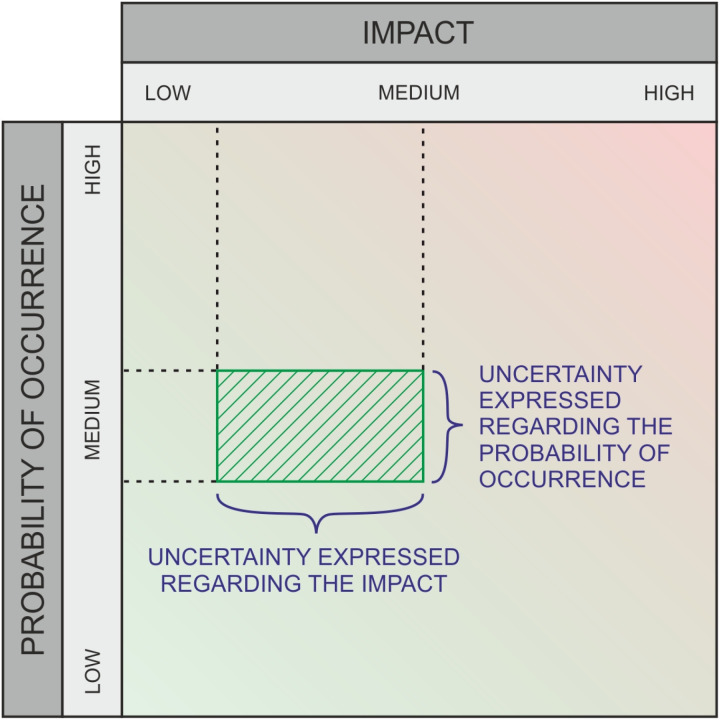
Graphical risk assessment method.

This method is popular for its advantage of being easy to implement, easy to explain and to communicate, as well as workable with vague or imprecise information. Also, it is applicable to “opportunities”, i.e., the positive counterpart to “risks” where possibilities are likewise rated in terms of impact (*gains*) and likelihoods (see Fig 3). However, a limitation of simple risk matrices is the difficulty in expressing and representing uncertainties, as experts always have to decide on a specific value for each assessment. Bubble charts are a useful graphical representation to extend risk matrices by letting a bubble correspond to a threat, with its x/y position marking the impact and likelihood, and the size of the bubble reflecting either the product of the two (the quantitative risk), or the uncertainty about either of the inputs. In [5], bubble charts were proposed in a modified form to serve as an “input system” for risk specifications, letting users draw rectangles (instead of bubbles), to specify impact, likelihood and uncertainty about both dimensions, by drawing a rectangle in an area that corresponds to the familiar risk matrix; see Fig 1).

**Fig 2:**
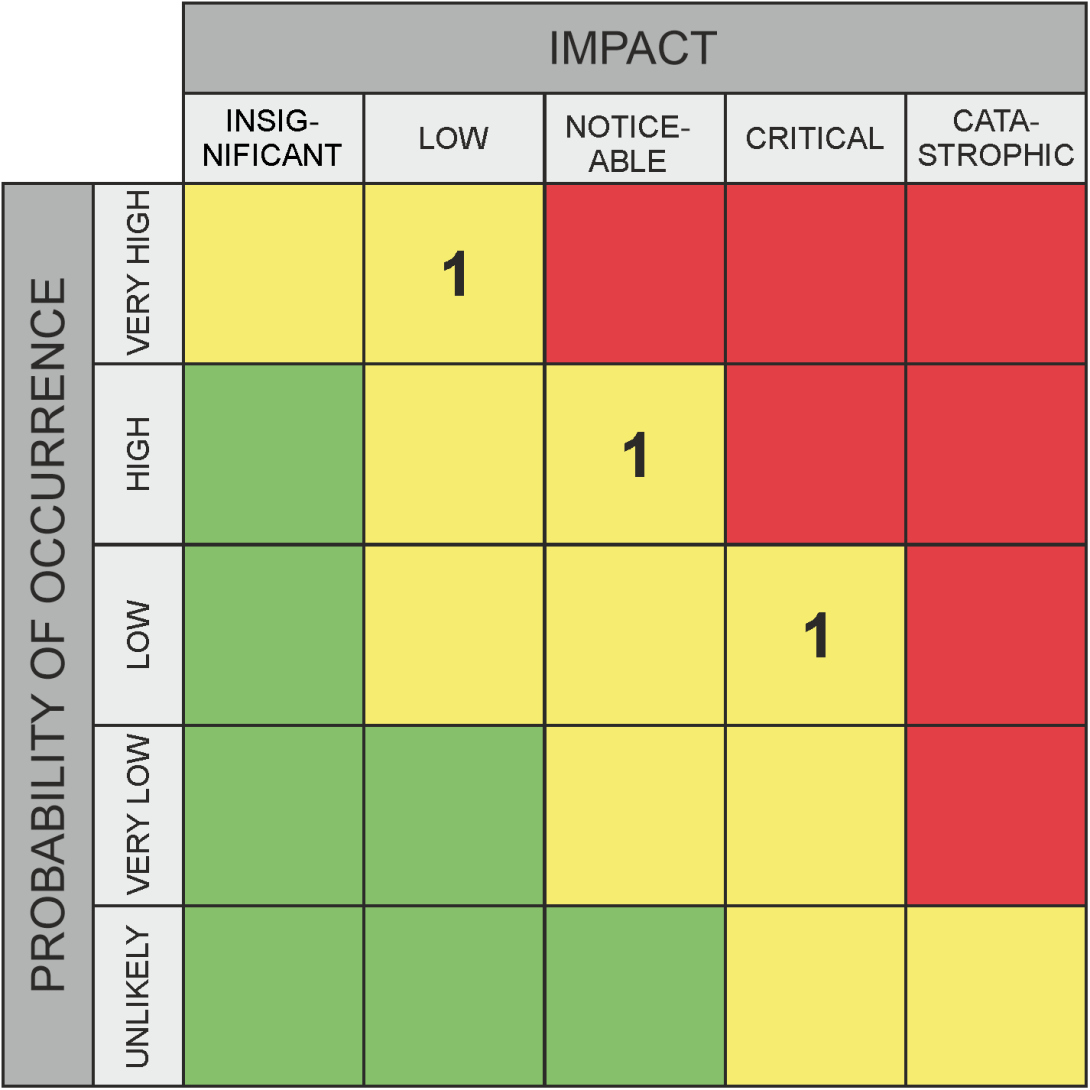
Classic assessment for risks – example.

**Fig 3:**
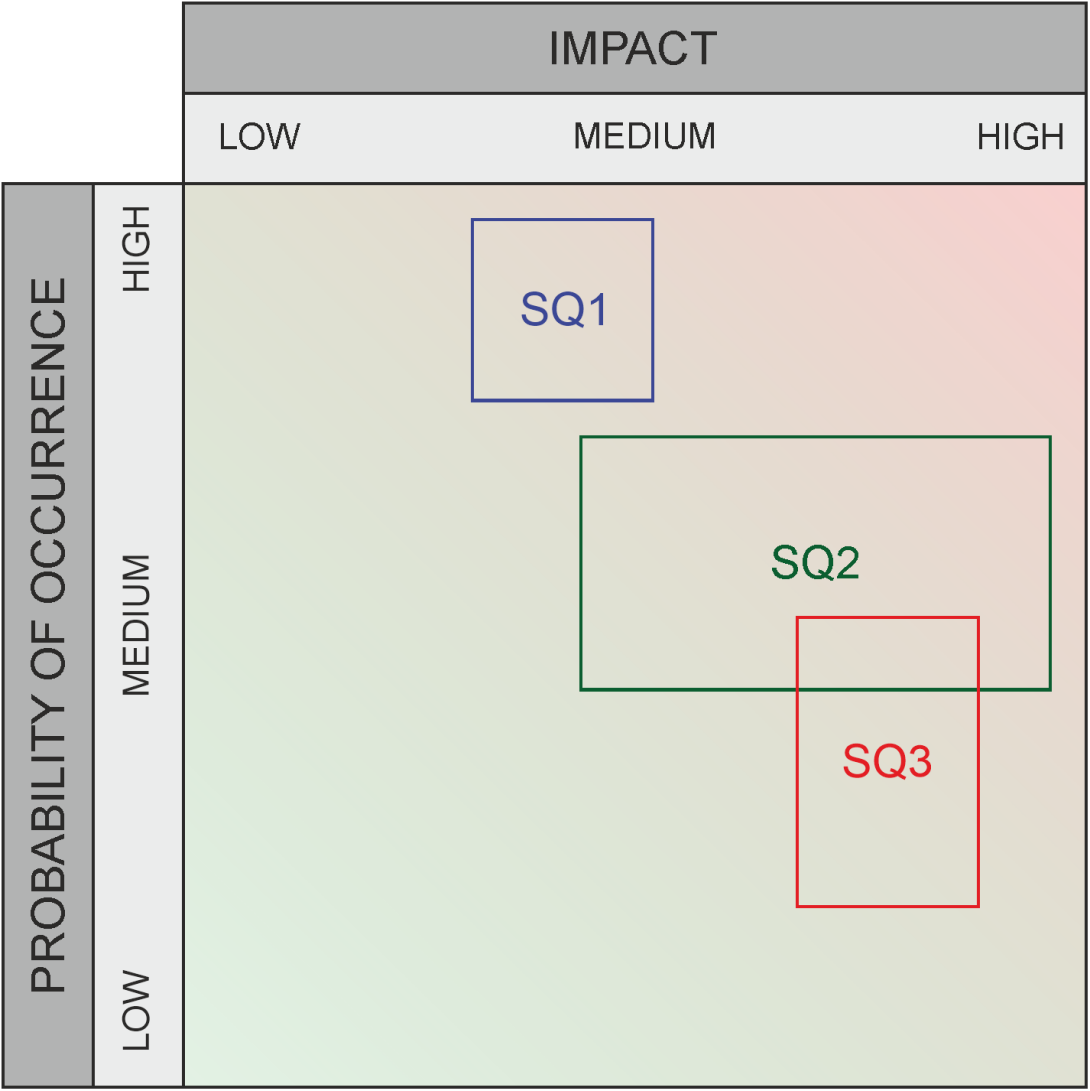
Graphic assessment for risks – example.

This would allow both the probability of occurrence and the impact to be defined over a range, rather than in stages as was originally the case. In the presented approach, the various assessments are subsequently aggregated.

**Fig 4:**
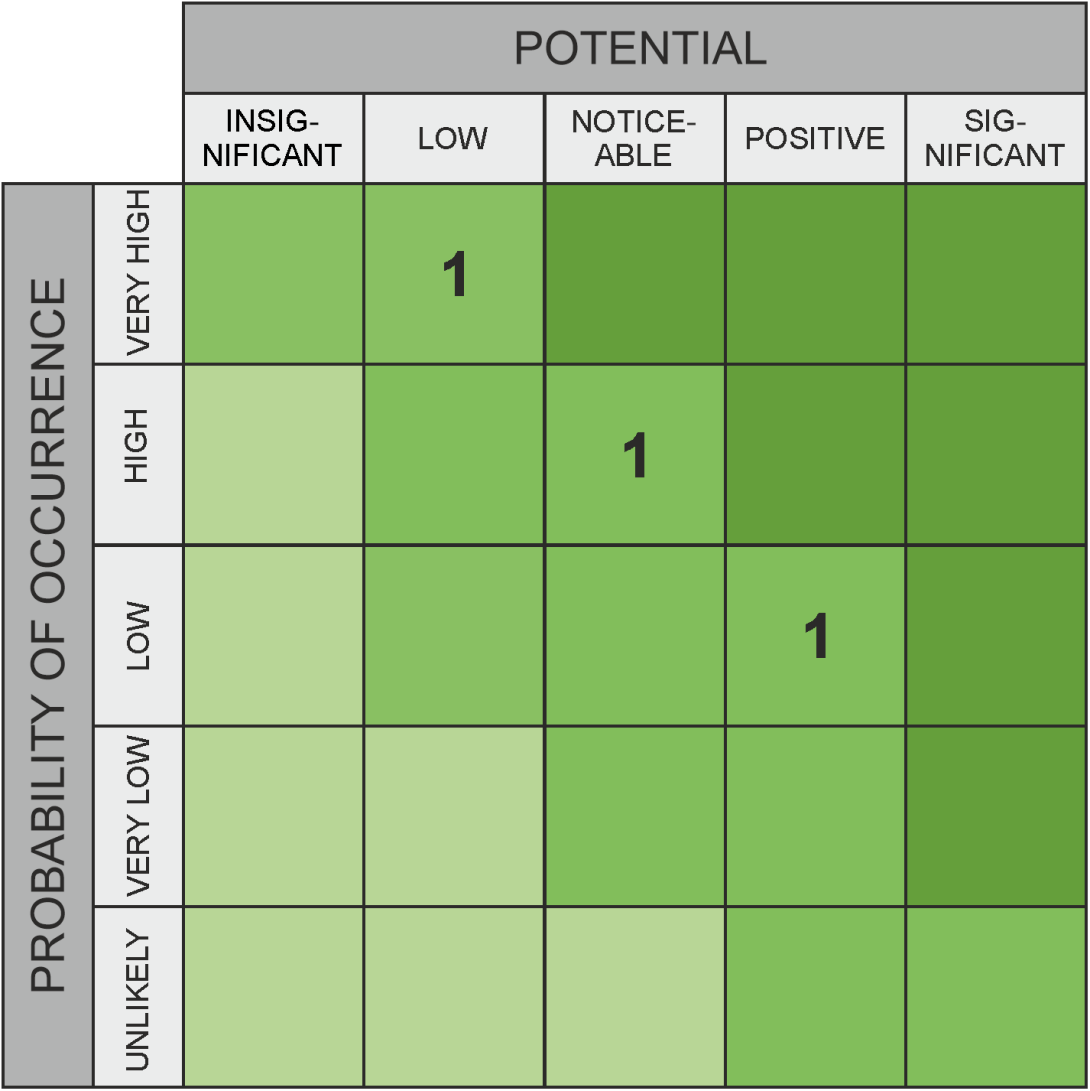
Classic assessment for opportunities – example.

**Fig 5:**
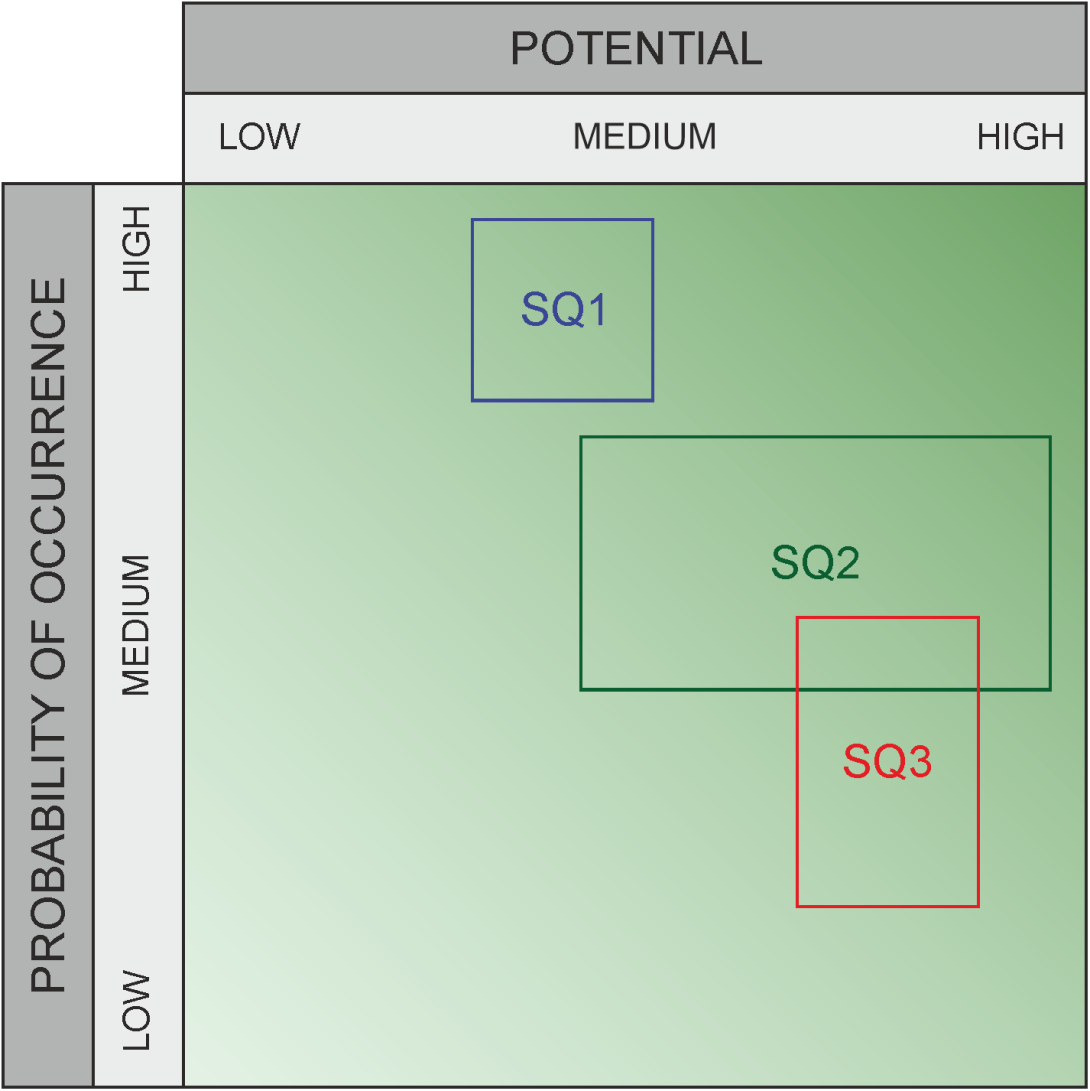
Graphic assessment for opportunities – example.

Recent research has shown that, for example, it is a significant challenge for healthcare experts to understand the interactions between different organizational areas and external factors amidst the increasing complexity of technical systems [7,8]. This complexity makes it difficult to clearly assess the likelihood and impact of various threats. To average out the subjective variations in data about risks (impact and likelihood category specifications), many experts are usually asked to rate the same threat; marked with “1” in Figs 2 and 4, or with “SQ1, SQ2, SQ3” in Figs 3 and 5. The output variation is often due to strong interdependence between domains [9,10] (due to external factors that can induce risks despite all “local” risk management), and influenced by a person’s own professional background. For example, a risk assessment made inside a hospital needs to take into account the facility’s dependence on external suppliers and will to a significant extent depend on the awareness and professional background of the person making the assessment. For the traditional risk matrix, the final assessment is then compiled by averaging over all values, taking their median, or similar. For a method that outputs intervals rather than values, other aggregation methods are required, and this work studies several options in comparison to each other.

As part of this empirical research, the graphical evaluation approach will be qualitatively assessed through a survey. The study aims to develop a survey design that ensures adequate comparability, enabling a statistically supported evaluation of the applicability and usefulness of the alternative method. Risk and opportunity assessments frequently rely on matrices. Figs 2 and 4 illustrate examples with three ratings each. These matrices divide both the probability of occurrence (X-axis) and the impact (for risks) or potential (for opportunities) (Y-axis) into several categories, typically ranging from three to five. These categories represent ordinal scales. A key limitation of this approach is that it requires selecting specific levels on both axes, even when the evaluator is unable to make a precise judgment. In the absence of a clear opinion, respondents may default to the middle category if available. To mitigate this, an even number of categories is sometimes used to enforce a directional tendency. However, this approach still lacks a means to explicitly represent uncertainty. To address this issue, alternative survey methods have been developed to incorporate uncertainty into assessments. Some approaches use circles or rectangles to express variability. One such method, which allows respondents to select a relevant region using a square, enables the representation of different degrees of variance – thus incorporating uncertainty – along both the X and Y dimensions. This method, which captures uncertainty in probability of occurrence and impact or potential, is the focus of our research, as detailed in the next section.

## 2. Related work

Risk assessment and the associated uncertainties have been extensively studied in various fields, including cybersecurity, business continuity management [11,12], critical infrastructure protection, and decision sciences. Numerous studies have highlighted the challenges of integrating subjective expert opinions, handling incomplete or contradictory data, and ensuring methodological robustness in risk analysis. Over the years, several frameworks and methodologies have been developed to enhance the accuracy and reliability of risk assessments while accounting for inherent uncertainties.

This section provides an overview of previous research on risk management and uncertainty (Section 2.1) as well as alternative methods of risk analysis (Section 2.2). Particular attention is given to approaches that incorporate uncertainty into risk assessments and to the various aggregation techniques proposed for combining expert opinions.

### 2.1 Risk management and uncertainty

Risk management [13] is a matter of working with subjective and uncertain data. Critical infrastructures are particularly challenging in this regard, as uncertainty arises not only from a lack of details about the infrastructure itself but also from opaque dependencies on other infrastructures and the diverse professional backgrounds of the experts providing the data. New approaches to handle subjectiveness and uncertainty are thus constantly being researched in this regard [14]. The consideration of uncertainty plays an essential role in the fields of artificial intelligence, security and risk management [15–17] and is addressed in many theoretical as well as practical papers. There are various areas of risk management (e.g., environment, health, critical infrastructure) in which individual causes of uncertainties [18] exist and methods are being researched to compensate, consider or circumvent these uncertainties [19,20]. Examples of these methods include fuzzy logic, probability theory, and robust decision-making [21], which have been widely discussed in research papers, particularly in the context of high uncertainty [22]. The two most important reasons for considering uncertainty are, that it is necessary for decision making to know the presence of uncertainty in order to make a valid decision, and that you risk losing credibility and trust if you do not deal openly with uncertainty [23]. Therefore, especially in complex contexts, it is important to be aware of the importance of uncertainty for the assessment of risks and the decisions derived from it [24]. There are several reasons for the occurrence of uncertainties in risk analyses [25] (e.g., lack of information or knowledge, abundance of information or knowledge, conflicting nature of pieces of information and data, measurement errors, linguistic ambiguity, subjectivity of analyst opinions), which in turn are studied in many scientific papers to better understand and evaluate the occurrence of these uncertainties [26,27]. Essentially, a distinction is made between two different types of uncertainties [25], Aleatory uncertainty (i.e., due to inherent randomness) and Epistemic uncertainty (due to lack of knowledge) [28]. For expert specification, declaring uncertainty is the primary concern, while the source of uncertainty becomes relevant at a later stage during risk control. This distinction is crucial, as epistemic uncertainty can potentially be reduced through additional knowledge acquisition, whereas intrinsic randomness (aleatory uncertainty) may remain beyond control. To better manage epistemic uncertainty, methods that provide more detailed information are preferable. The graphical method enhances this process by improving the representation of available expert knowledge and enabling structured input and retrieval of this knowledge for subsequent presentation and analysis. This is intended to provide decision-makers with sufficient information about existing uncertainties and to illustrate them in a transparent and comprehensible manner in order to be able to derive correct measures and to better understand interrelationships [29].

### 2.2 Methods for risk analysis

For the analysis of risks, risk matrices are used in most cases in the context of risk surveys, whose application is critically questioned in various scientific contributions [30,31]. The factors often criticized in this context are the neglect or disregard of uncertainties and the disregard of psychological influences on the estimation of risks. In the recommendations for the use of risk matrices (see [32], as well as section 1), approaches for the consideration of uncertainties have already been indicated. In the field of risk management, a variety of different methods have been studied that analyze psychological factors in the assessment of risks. In the survey, some of the psychological factors that are discussed in the paper [33] will also be applied. Furthermore, there are various research approaches that represent alternative methods to the so-called “classical” risk matrix presented in the introduction, with the purpose of being able to include relevant influencing factors [34–36]. The risk assessment process itself has also been criticized and there are recommendations to redefine the process from the perspective of using more detailed information and better solutions for the assessment tool itself. [37] Much of this research and the methods developed for it, have not been evaluated in detail or have been applied only to case studies [38–41]. In the field of critical infrastructure, there has not yet been a direct comparison between a conventional risk assessment method and those that also allow the representation of fuzziness. A recent scientific study explicitly points out the importance of further experimental research in risk matrices to find better ways to represent uncertainties in risk assessments [42]. Based on a continuous probability-consequence diagram, in which boxes express uncertainties in the probability of occurrence and impact of risks [43], a method for capturing risk assessments with the mentioned fuzziness was developed [44]. A wide variety of statistical procedures can be applied to the results of a survey using this risk assessment method. These procedures have the approach of correcting the data accordingly and allowing the data to be aggregated [5,45].

## 3. Aggregation methods

While classical risk assessment, which delivers numerical values or categorical ratings, offers various well-established methods for aggregating expert opinions – such as averaging, median calculation, and opinion pooling, among others [6,46] – comparable methods for aggregating graphical risk assessments are lacking. To address this gap in the literature, we propose several potential approaches. Table 1 provides an overview of the aggregation approaches commonly used in classical risk matrices and the aggregation methods proposed in this paper for the graphical survey method, offering a clear comparative summary of their main characteristics.

**Table 1:** Comparative overview of different aggregation methods in the classical and graphical case.

	Method	Brief description	Uncertainty of individual opinions considerable?	Similar/ overlapping opinions considerable?	Result	Possible issue/limitation
Traditional aggregation methods, based on classical risk matrix	Majority Vote	The most frequently selected rating prevails, reflecting the opinion of the majority.	No	yes	Most frequent risk category	Ignores extreme risks
	Averaging	Calculates the arithmetic mean of all risk ratings to provide a balanced assessment.	No	yes	Average risk category	Potentially underestimating extreme risks.
	Median	Uses the median value to reduce the influence of outliers.	No	partly	Median risk category	can miss multiply indicated (and hence potentially relevant) extremes
	Maximum Category	Takes the highest risk category as the final rating, emphasizing worst-case scenarios.	No	not generally	Highest risk category	Overly conservative, may inflate risk perception
	Opinion Pooling	Combines multiple expert assessments, often applying weighted adjustments.	No	yes	Appears precise but lacks uncertainty considerations in the classical risk matrix.	Depends on expert reliability
Alternative aggregation methods based on the graphical risk assessment method	Areas	Combines participants’ selected areas in a 2D risk matrix.	Yes	partly	Aggregated area covering all selections	Can dilute outliers, less precise individual assessment
	Weighted Areas	Extends area aggregation by emphasizing frequently selected regions.	Yes	yes	Weighted aggregation of frequently selected areas	Can overemphasize clustered selections, reducing sensitivity to outliers
	Center	Computes the mean center of selected areas for risk aggregation.	No	not generally	Mean center point represents aggregated risk assessment	Loses distribution details, simplifying complex assessments
	Center to Grid	Maps area centers to a 5 × 5 risk matrix grid.	No	not generally	Mapped risk category based on center point location	May misrepresent risks by reducing variability
	Reached Grid	Considers all grid cells covered by a selection instead of just the center.	No	yes	All covered grid cells contribute to risk aggregation	Might inflate risk perception if large areas are selected
	Opinion Pooling	Merges expert opinions using weighted averaging, adjusting for uncertainty.	Yes	yes	Consensus-based risk value using weighted expert opinions	Highly dependent on expert weighting and initial assumptions

Since the graphical method delivers rectangles rather than numbers, an “aggregation” will hereafter result in a (probability) distribution and not a crisp number. Although the classical method gives crisp values, distributions can still be constructed from the absolute frequencies of individual responses over the finite set of possible answers. This allows us to compile an outcome distribution for the classical method as well, enabling a comparison of the graphical and classical methods, despite their results having inherently different “data types.” Additionally, information loss due to averaging, median calculations, or similar methods is reduced, since the distribution can encapsulate more information.

**Fig 6:**
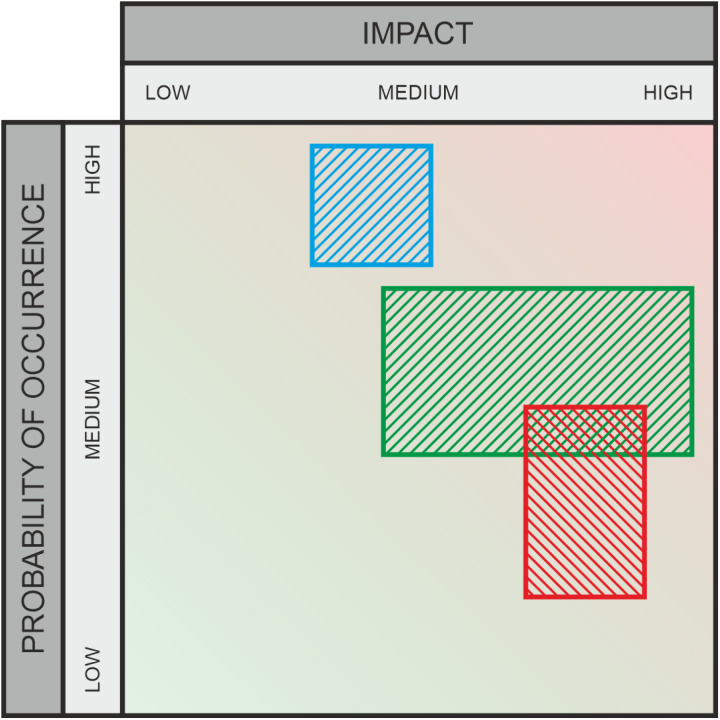
Graphic assessment – areas.

**Fig 7:**
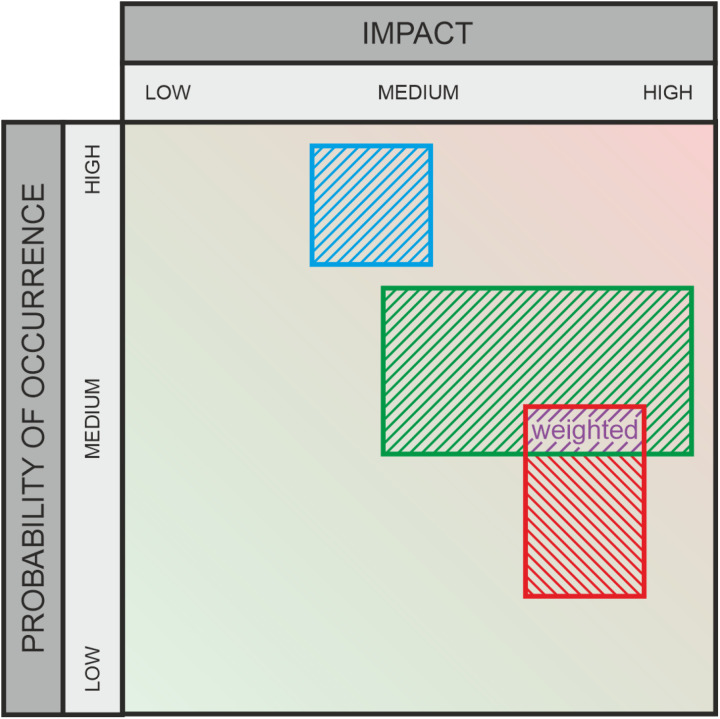
Graphic assessment – weighted areas.

**Fig 8:**
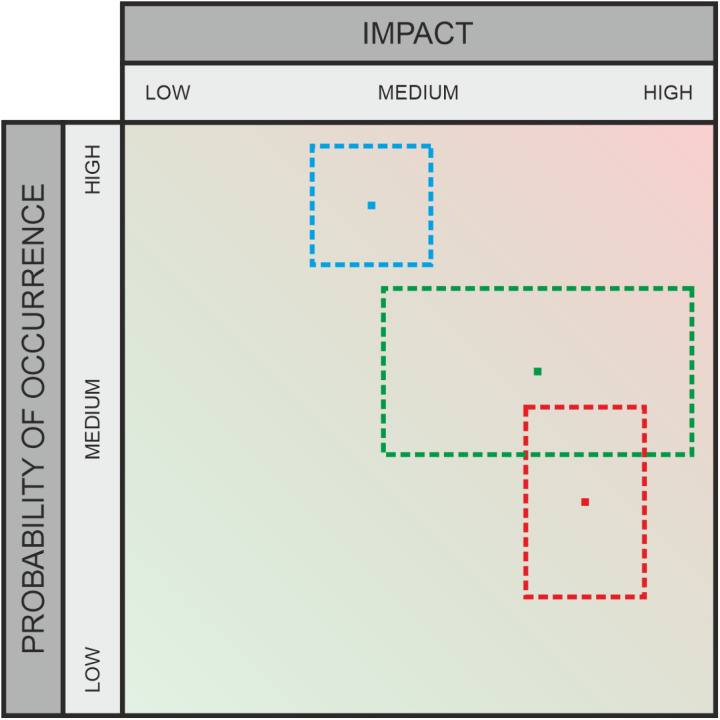
Graphic assessment – center.

**Fig 9:**
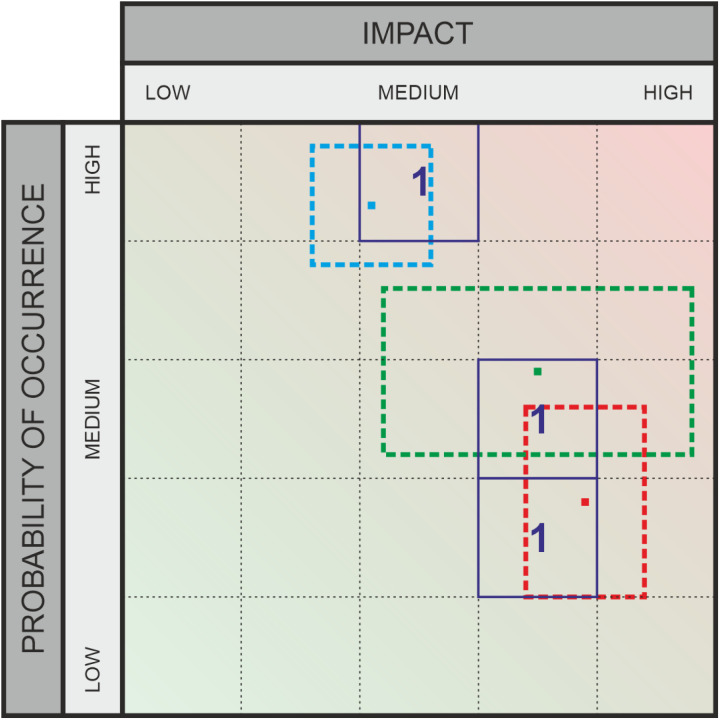
Graphic assessment – center to grid.

**Fig 10:**
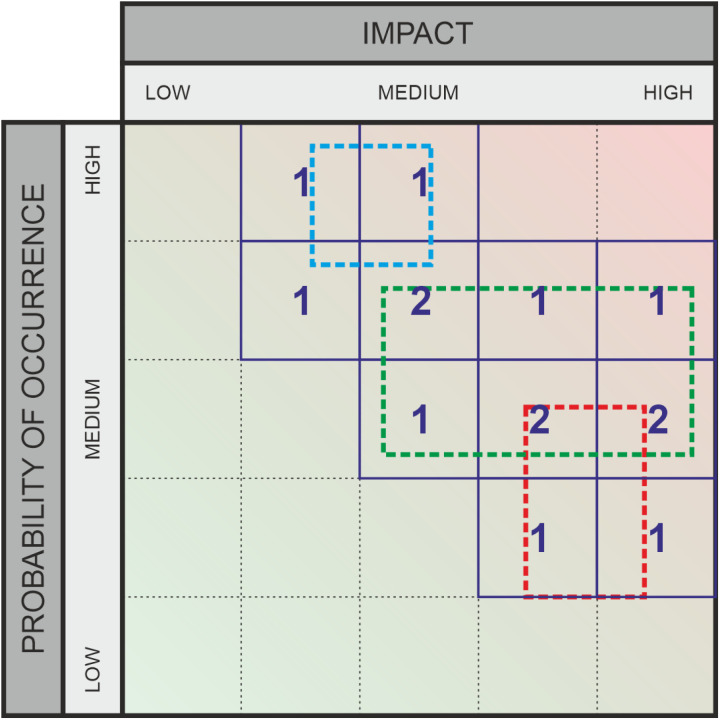
Graphic assessment – reached grid.

**Fig 11:**
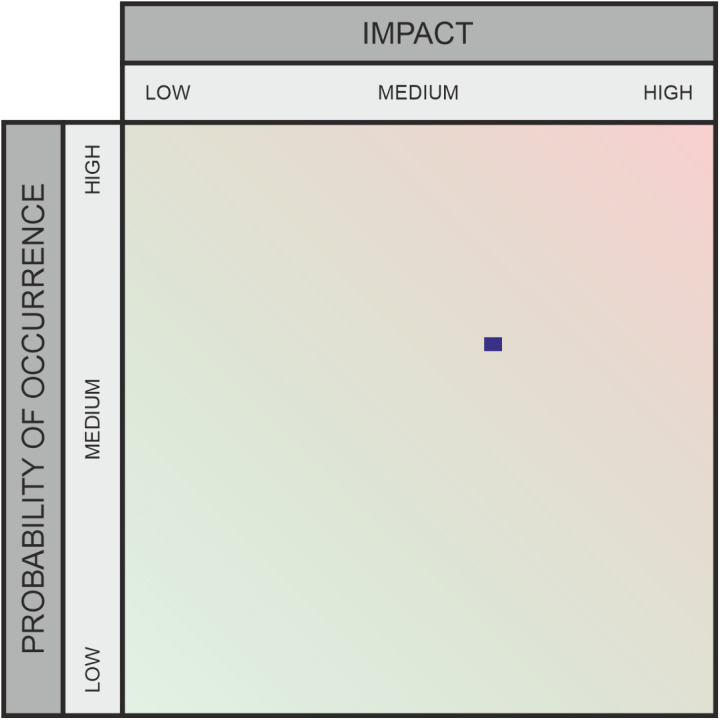
Graphic assessment – opinion pooling.

We propose a set of different aggregation methods, apply them to the data obtained from the risk assessment, and compare the results. The following paragraphs provides a comparative overview of the characteristics and distributions of both the classical and the proposed aggregation methods. To analyze the results of the graphical method, for example as in Figs 3 and 5, we have selected the following different aggregation approaches (Figs 6–11), which are then compared with the classical method. All selected methods allow for a detailed analysis of the distribution, scattering dimensions, and visualizations of risk and chance preferences across two independent dimensions – impact and probability of occurrence. The aggregation process is only used for the graphical assessment because the classical method delivers a more straightforward and less complex evaluation that does not require additional aggregation, as it does not account for uncertainty.

### 3.1 Aggregation by areas (Fig 6)

This method is used for the aggregation of assessments made by multiple participants within a two-dimensional evaluation space. The approach of this method is to examine the spatial preferences of the participants by separately evaluating their selection ranges (respective segments) along the X-axis (Impact or potential) and the Y-axis (Probability of Occurrence) of the evaluation space. The aggregated value is a distribution supported on the union of all the areas, and with a mass proportional to how often a region was covered by an individual’s section. It represents the combined assessments of all participants.

### 3.2 Aggregation by weighted areas (Fig 7)

This aggregation approach extends the previous “Aggregation by Areas” method for analyzing risk assessments in a two-dimensional evaluation space by introducing additional weighting for areas consistently selected by participants. This method aims to highlight consensus areas within the selected assessments by assigning increased importance to overlapping selections. The methodological innovation lies in the differentiated consideration and analysis of spatial preferences along the X and Y axes, complemented by a weighting function that quantifies agreement among participants.

The ranges on the X and Y axes are normalized by calculating the normalization range as


$$1-\;\frac{\left(\max.range-\min.range\right)}{100}.$$

For each user, weights are calculated based on the normalized ranges of their responses as follows: Responses that overlap with other responses receive a higher weight, as they have greater significance for the overall evaluation. Based on these weights, a distribution of values along the X and Y axes is generated for each user. These distributions are then aggregated in steps of 0.25 by summing the corresponding weights. Finally, the aggregated data is grouped by scale values, where both the total sum of the weights and the normalized probabilities are calculated. This method highlights consensus areas by assigning greater importance to overlapping selections and provides a detailed spatial analysis of risk assessments along the X and Y axes.

### 3.3 Aggregation by center (Fig 8)

This method computes the center point of each area in the X and Y dimensions (i.e., for Impact and Occurrence) and calculates the arithmetic mean of these center points across all participants to derive the final aggregate value. By reducing the individual user selections to a single midpoint on both axes, this approach allows for a simplified yet accurate representation of risk preferences within the studied group. A key feature of this approach is the simplification of data representation, which, however, does not explicitly take into account the inherent uncertainty and variability of the original user selections.

### 3.4 Aggregation by center to grid (Fig 9)

In this aggregation method, the entire graphical selection area is evenly mapped onto the 25 fields (5 x 5 categories) of the classical risk or opportunity assessment method. The aggregation result is the grid cell (as in the classical method) in which the center of the respective rectangle is located. Thus, for a user evaluation through a rectangle, a center point is established, and an associated field of the classical method is determined. This aggregation procedure leads to a loss of information regarding uncertainties in graphical evaluations. It corresponds to the hypothesis of a user providing only a range, but no further information on where within this range the actual risk would be, thus expressing a “uniform distribution”. Its center is then the “expectation within the specified range”, i.e., corresponds to a crisp risk specification based on the user’s uncertainty. This method, at the same time, makes the graphical outcome comparable to the classical outcome (for subsequent tests).

### 3.5 Aggregation by reached grid (Fig 10)

Similar to the ‘Center to Grid’ method presented above, this aggregation method maps the entire graphical selection area onto the 25 fields (5 × 5 categories) of the classical risk or opportunity assessment method. However, all fields of the classical method covered by the selected rectangle are considered in the evaluation. Depending on the size and overlapping area of the rectangle, one or more classical fields may be considered in the evaluation. All fields covered by the selected rectangle across multiple assessments are aggregated, resulting in a distribution ranging from one to five for both impact (or potential) and probability of occurrence. Compared to the previous method, this approach is more specific in identifying regions where the actual risk is more likely to be, resulting in a ‘non-uniform’ distribution based on differing expert opinions.

### 3.6 Aggregation by opinion pooling (Fig 11)

The Opinion Pooling method described in [5] aims to aggregate multiple expert opinions into a single risk value by a weighted average, with weights inversely proportional to the variance (certainty) of the respective person. The challenge is to find consensus, especially when data is sparse and expert risk assessments vary significantly. Traditional communicative methods like the Delphi technique or time-consuming discussions often fail to reach consensus. Instead, mathematical pooling functions and formulas are used to merge opinions into a single value. This approach is known as mathematical Opinion Pooling and has a long tradition in statistics, both in combining forecasts and in decision-making. A simple and straightforward Opinion Pooling method is averaging all values, i.e., calculating the arithmetic mean or median. This method is widely used and often applied uncritically, although it has significant drawbacks, especially its susceptibility to outliers (where the median is more robust, but still not entirely insensitive). Extreme data points can significantly shift the aggregated value, thus distorting the final result. Moreover, when aggregating data, robust approaches as well as the detection and correction of outliers should be considered, especially if the dataset is small. The different levels of experience and knowledge of individual experts, as well as their certainty or uncertainty regarding their risk quantification statements, should also be taken into account. As a solution, the authors of [5] propose an intuitive iterative Opinion Pooling scheme that considers all the aforementioned aspects. This approach is viewed as a lossy form of data aggregation as opposed to lossless aggregation, where the complete data defines a full distribution. The iterative scheme is based on weighting opinions, adjusted over several rounds until consensus is reached. Opinions are smoothed and updated based on a discrete inverse distance kernel function, following the rules of Bayesian updating. This method considers not only data smoothing but also each expert’s confidence in their risk judgment, giving more weight to risk assessments with high certainty than those with low certainty.

## 4. Research questions

The diversity of risk assessment methods, together with their common lack or insufficiency of handling uncertainty, motivates our main research questions:

RQ1 **How to summarize data?**

How can the subjective risk and opportunity assessments of several experts, who use either a classic or graphical assessment method, be aggregated into a final risk value?

We dedicate Section 4 to an answer to RQ1.

RQ2 **Differences between aggregation methods?**

Which data aggregation methods (reviewed in Section 4) lead to statistically similar or significantly different aggregated risk and opportunity assessments?

The examination of these methods is directly linked to the analysis of their individual results and performance. Sections 4–7 provide answers to RQ2. The second question implies a number of statistically testable hypotheses (e.g., the Kolmogorov-Smirnov test). Based on the defined risk and opportunity scenarios (84 in total, as explained in Section 5), we assume that the results obtained from the various aggregation methods of the graphical analysis will be identical to those derived from the classical method. Section 5 also provides a detailed explanation of the individual statistical tests used in this analysis.

RQ3 **Differences between summary statistics of risks and opportunities?**

Is there a significant difference between the results of the method comparison for the individual scenarios of risks and opportunities?

Section 8 provides an answer to RQ3.

The third research question is whether the results of the first research question differ significantly when comparing risk scenarios to opportunity scenarios. In Section 9, we summarize and discuss the complete set of results.

## 5. Application of statistical analyses to the methods

A main objective of this work is understanding whether the graphical method delivers substantially different results or results of different quality. To empirically measure such differences, we applied statistical tests to identify differences in distributions (here, Kolmogorov-Smirnov and Wasserstein distance) of the respective data (gathered by classical or graphical means). This enables a statistical interpretation of the results and thus a direct statement about the significance of the differences between the distributions or methods, based on p-values (which is a stronger approach than measuring similarity using simpler non-parametric methods like correlation). Our choice of the Kolmogorov-Smirnov method to compare distributions is justified for several statistical and methodological reasons:

- Non-parametric test: the Kolmogorov-Smirnov test does not assume any specific distribution. This is particularly important because “risk” is not a physical quantity that can be objectively measured (and thus does not have a “natural” distribution). Without clear information about the data distribution and the distributions of interval centers, the aggregated data is unlikely to correspond to any common assumptions (e.g., normal distribution).
- Sensitivity to differences: The test measures the maximum difference between the cumulative distribution functions of two samples. This makes it particularly sensitive to differences in central tendency, variability, and shape of the distributions. Since the selected methods generate entire distributions over a range of 0 to 100, a test that captures differences in the entire distributions is ideal.
- Applicability to different data scales: The Kolmogorov-Smirnov test can be applied to both ordinal and interval-scaled data. This makes it possible to compare the effectiveness of different aggregation methods without artificially adjusting the data.

An additional test using the Wasserstein metric was conducted as a complementary measure to verify the robustness of the results, while the primary conclusions are based on the Kolmogorov-Smirnov test.

Each aggregation method provides a distribution for both the impact and the probability of occurrence. These distributions are shown in the following example for each method for both axes as histograms together with the associated scatter measures. The Kolmogorov-Smirnov distance was therefore chosen to measure the extent to which the distributions differ per scenario and respective method. Alternative methods such as polyserial correlation, were considered. The classical method provides an ordinal variable with the ratings on an ordinal scale {1 < 2 < 3 < 4 < 5}, which is required for the polyserial correlation. For polyserial correlation, however, the second variable must be metric, which is not the case in our setting. The graphical method provides intervals for impact and probability of occurrence instead of scalar values, which makes direct correlation with the classical method difficult. A straightforward resolution would be using the interval centers of the graphical method, but this would practically abandon the additional information that the graphical method delivers. However, since the different aggregation methods provide entire distributions over the entire range of values, they should be compared using a statistical measure that evaluates the similarity of distributions, such as the Kolmogorov-Smirnov distances. These distances are supplemented with a p-value in the course of the analysis. Instead of results such as “‘Method A’ correlates significantly/non-significantly with ‘Method B’”, the Kolmogorov-Smirnov test provides results of the type “The results of ‘Method A’ and ‘Method B’ differ significantly/non-significantly”. Due to the difficulties described above, the data and aggregation methods of the graphical method do not fit well with correlation approaches, which is why an approach must be chosen that compares entire distributions.

For a pairwise comparison of the seven methods (including the classic assessment) of the previous section, we thus run a total of 2·(7·7−7)=84 statistical tests (comparing every method with every other method (7·7), excluding the comparison of a method with itself (hence “− 7”) and doing the comparison for both dimensions “impact” and “probability of occurrence”, hence the factor 2).

## 6. Methods and study design

**Fig 12:**
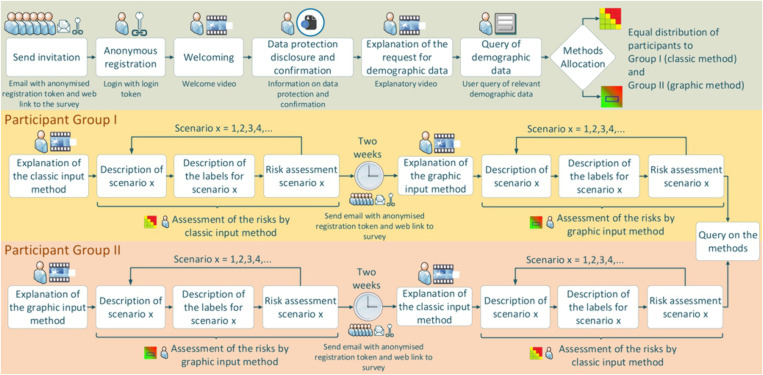
Detailed survey procedure.

The graphical risk assessment method described in the introduction should be compared to the traditional assessment method. The survey design was conceived so that respondents were randomly but evenly assigned to one of two groups. One group started the evaluation of the risk and opportunity scenarios with the traditional method, while the other group started with the graphical survey method. Each participant received all scenarios twice using both evaluation methods. Exactly fourteen days after the completion of the first collection phase, the participants received the same scenarios with the other method. Participants also had the option to skip a scenario they were unable to assess. In such cases, the skipped scenario was excluded from the repeated survey with the other method. Fig 12 illustrates the survey process in detail.

The research team had the opportunity to implement the defined survey process to obtain real and thus comparable data in the context of an actual critical infrastructure, namely the third-largest hospital in Austria. The hospital has a total of around 1,200 beds and 5,000 employees and is an important healthcare provider in the region, offering a wide range of medical services and specialties. Specifically, the survey was conducted during the planning phase of a complex new construction project for the hospital. The factor of ‘uncertainty’ plays a crucial role in such projects, due to their lengthy duration and inherent complexity. The survey participants were experts from this hospital who had been briefed multiple times on the categories of impact and probability of occurrence. These thresholds are well-established within the institution, ensuring a shared understanding of the rating scale among the participants. Additionally, the definitions of these categories were consistently accessible throughout the survey, allowing respondents to refer to them as needed. This consistent framework ensures uniform interpretation of both the severity of impacts and the probability of occurrence. Experts from various fields within the healthcare sector were consulted, all of whom possess extensive experience not only in overseeing and implementing hospital construction projects but also in managing the associated operational and organizational changes. Their expertise offers invaluable insights into the respective thematic categories and was therefore indispensable for the data collection in this study. The evaluation encompassed both risk and opportunity scenarios related to stakeholders, workflow, consequences, organization, environment, requirements, legal aspects, safety, technology, environmental and ecological factors, as well as unforeseen events within the project context. A risk management expert developed a catalog of project-relevant risk and opportunity scenarios. This catalog comprised a total of 86 scenarios, including 68 risk and 18 opportunity scenarios, which were subsequently incorporated into the survey. In subsequent analyses, responses and associated methods for risks and opportunities are separately examined due to significant variations in perception and assessment between these two categories. Furthermore, risks and opportunities fundamentally differ in their impacts and potentials. This separate consideration allows for a nuanced and precise analysis, contributing to a thorough and targeted investigation of the methods. Subsequently, the results from the risk and opportunity analyses can be compared.

The study was approved by the Ethics Committee of the State of Carinthia, Austria, prior to its commencement. All participants provided informed consent for the anonymous use of their data prior to participating in the online survey. The survey was administered via an online platform, and participation was only possible after explicit consent was given by ticking an agreement box on the introductory page of the survey. No minors or patients were included in the survey.

The recruitment period for this study took place from August 17, 2022, to December 9, 2022, to ensure a representative sample size and achieve the study objectives. Following this phase, research efforts focused on exploring aggregation methods to enable comprehensive data analysis and enhance the robustness of the results.

## 7. Results

The survey was completed in full by a total of 75 experts and covered 86 different scenarios. Each scenario was assessed using both the classical risk assessment method and the graphical method. In total, 11,732 assessments were collected across both methods. To illustrate the comparison of the methods, we have selected the following risk scenario from our survey in order to make it representative. By focusing on this scenario, we aim to illustrate the practical application of the different aggregation methods in comparison to the classical method.

“How do you assess the risk that can arise from the change of key persons (e.g., project management, users, planners, clients, persons responsible in individual areas, e.g., technology) in the project team? In particular, how would you assess the impact and probability of occurrence of the unavailability of key personnel and changes in the decision-making structures in the project?”

**Fig 13:**
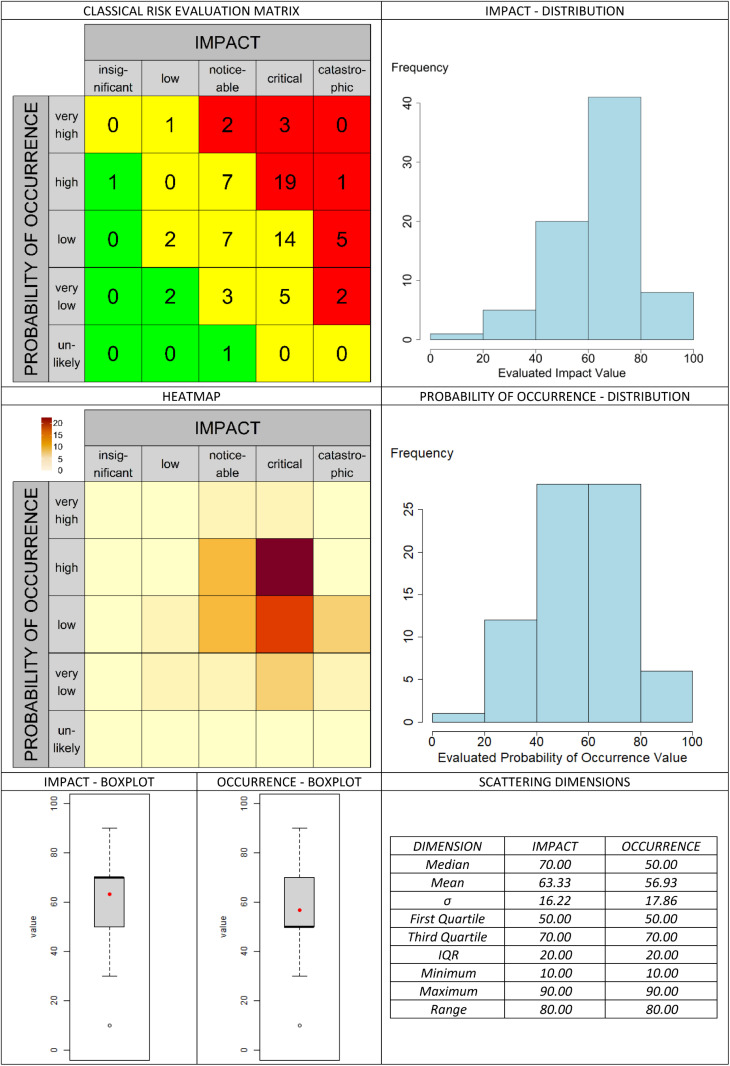
Classic assessment based on the example scenario.

**Fig 14:**
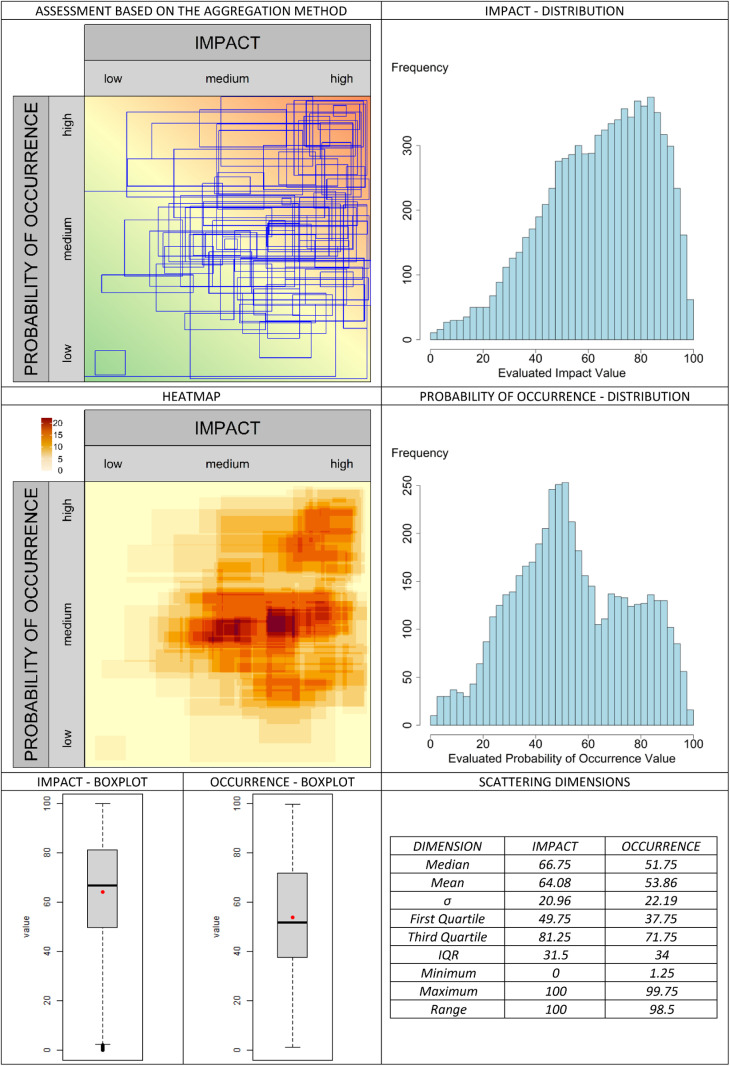
Aggregation method “areas” for the graphic assessment based on the example scenario.

**Fig 15:**
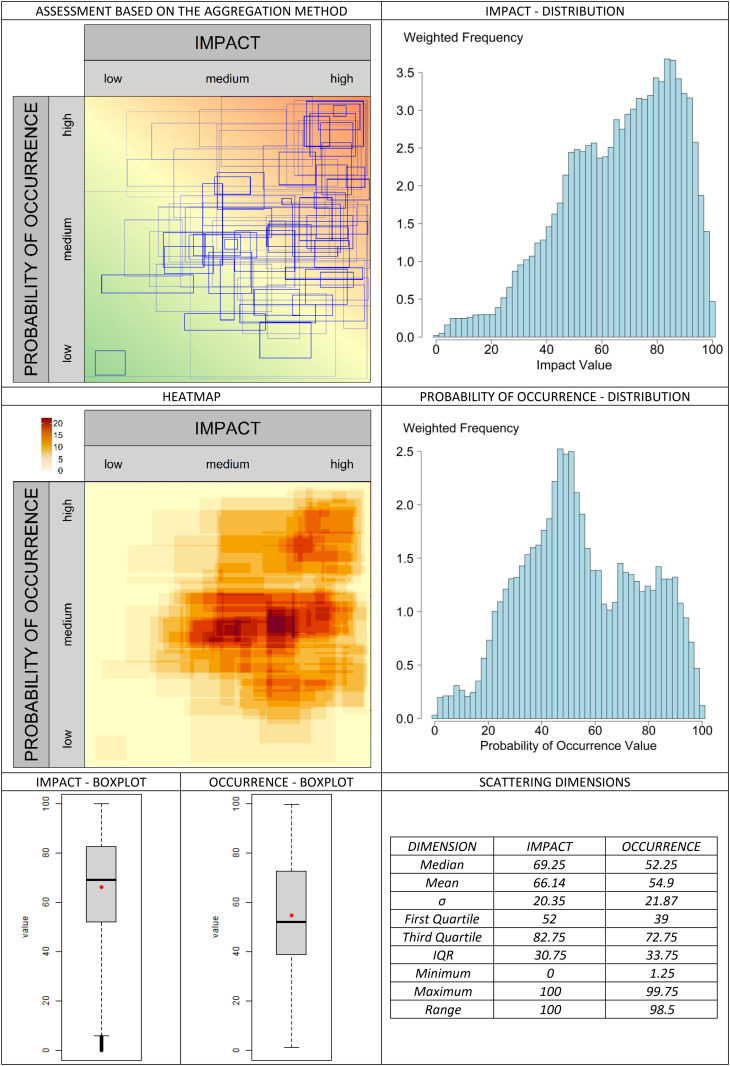
Aggregation method “weighted areas” for the graphic assessment based on the example scenario.

**Fig 16:**
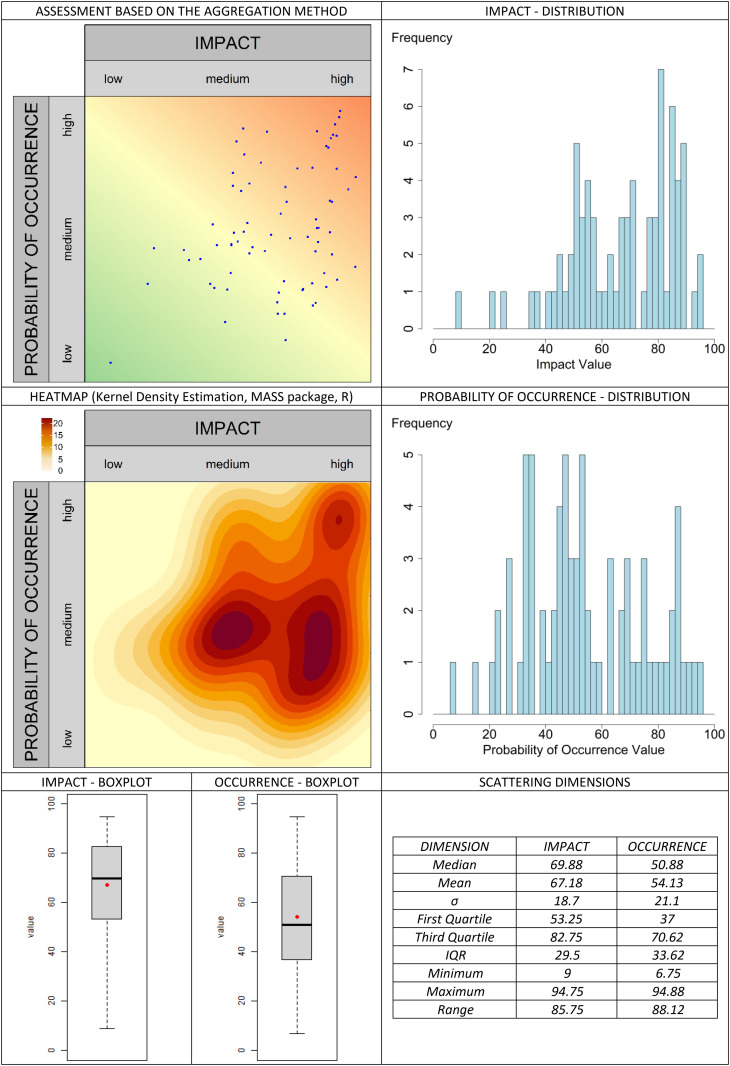
Aggregation method “center” for the graphic assessment based on the example scenario.

**Fig 17:**
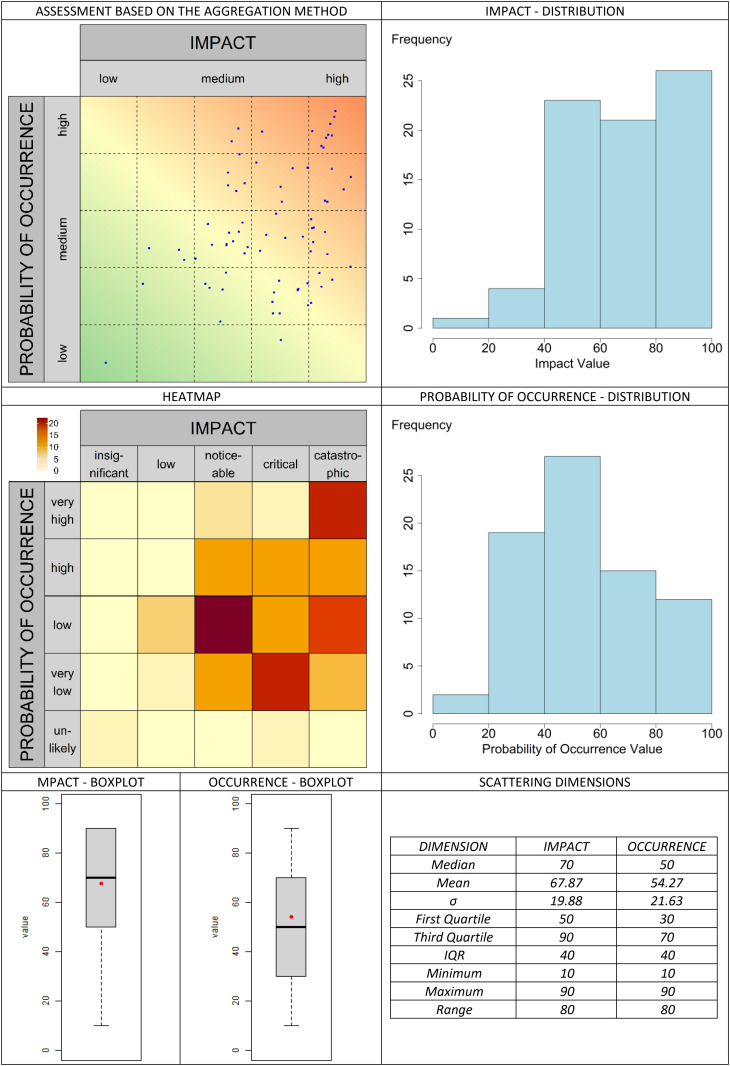
Aggregation method “center to grid” for the graphic assessment based on the example scenario.

**Fig 18:**
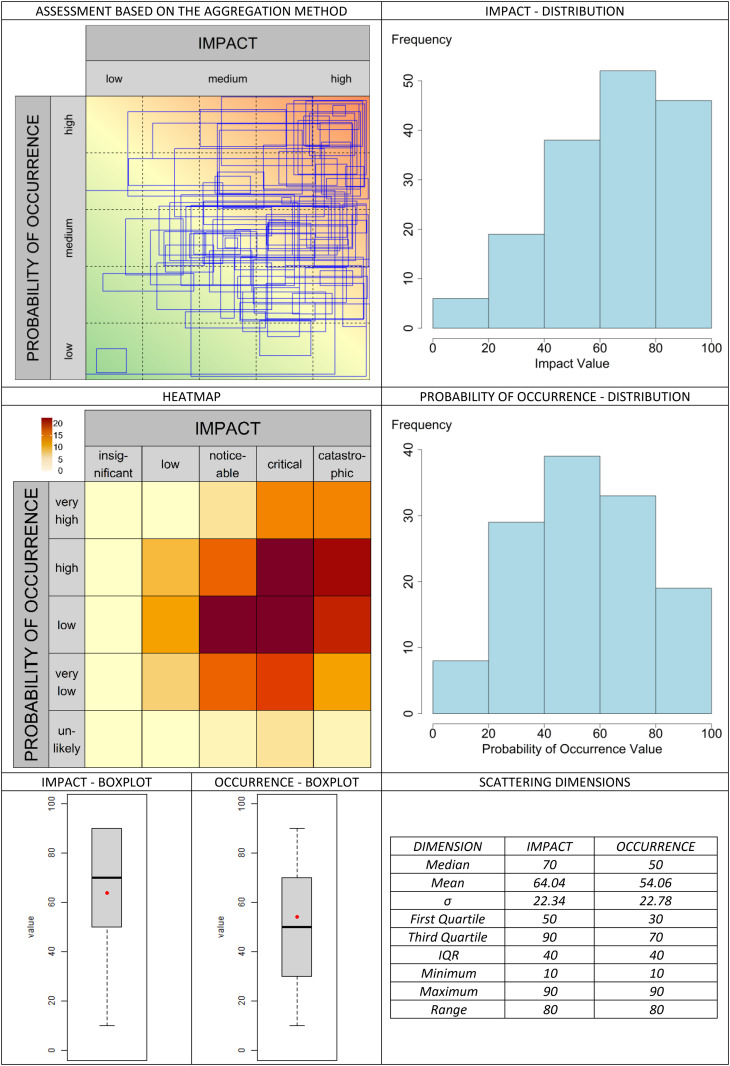
Aggregation method “reached grid” for the graphic assessment based on the example scenario.

**Fig 19:**
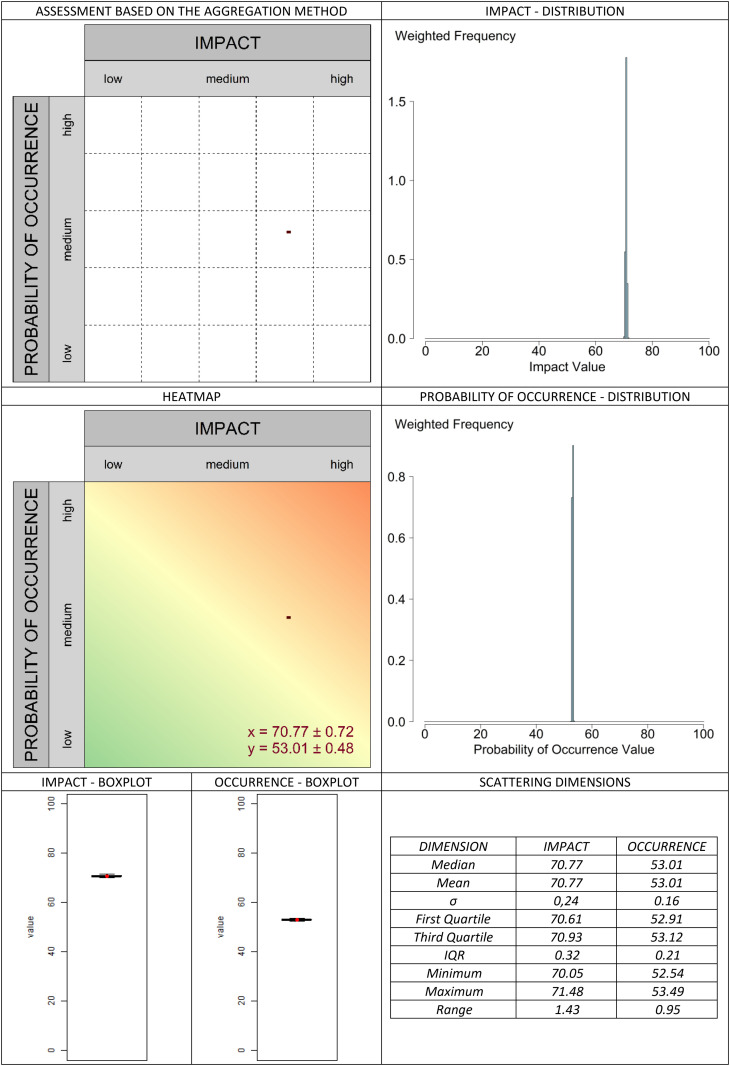
Aggregation method “pooling” for the graphic assessment based on the example scenario.

The classic valuation approach is presented in Fig 13. In the graphical assessment approach, the selected aggregation methods are compared in Figs 14–19. It is important to note that the axes in the graphical method are continuous, with the three labels (‘low’, ‘medium’, and ‘high’) serving merely as visual aids for the user. They should not be misunderstood as marking (discrete) categories as in the classical case. A methodological advantage over the classical method is that some aggregation methods are not constrained by the fixed structure of traditional risk matrices (e.g., 5 × 5 grids). This allows for a finer binning of the data (see Figs 14–16), which generally results in better information preservation compared to coarser classifications.

The results regarding the impact and probability of occurrence were presented in a standardized format using various visualization techniques: evaluation matrices, heat maps (showing how many people have selected certain regions in the impact/ probability of occurrence plane, corresponding to the respective image located above in the table), box plots, and distribution diagrams (showing the accumulation of choices along the range from 0 (min) to 100 (% max) of the probability of occurrence/impact scale), as well as some standard summary statistics (“Scattering dimensions”).

## 8. Data analysis

The distributions of the various methods were tested against each other for both impact and probability of occurrence using the Kolmogorov-Smirnov method. As an example, only the data tables for the risks are presented to address Research Questions 1 and 2. The average distances between the methods for all risk scenarios are shown in Table 2. This table is structured as a matrix, with the values for the method comparison regarding impact presented in the upper right area and the values of the method comparison regarding probability of occurrence presented in the lower left area. For example, the comparison of the “classic” method to the “centertogrid” method on the “Impact” dimension is found at the x/y location “classic/centertogrid” with a value of 0.14, while the corresponding comparison of the same methods for ‘probability of occurrence’ is found at the transposed x/y location “centertogrid/classic” with a value of 0.18. To validate the robustness of the results, we additionally applied the Wasserstein test, which confirmed the findings obtained with the Kolmogorov-Smirnov test. The Wasserstein test yielded the same conclusions regarding the statistical differences between the methods, further supporting the reliability of our comparative analysis.

**Table 2:** Comparison of methods using the averaged Kolmogorov-Smirnov distance of all risk scenarios.

aggregation method		classic	area	center	centertogrid	reachedgrid	weighted	pooling
		IMPACT						
classic	OCCURRENCE		0.30	0.31	0.14	0.15	0.30	0.66
area		0.32		0.11	0.23	0.18	0.04	0.60
center		0.34	0.08		0.24	0.23	0.08	0.57
centertogrid		0.18	0.21	0.22		0.09	0.21	0.60
reachedgrid		0.17	0.18	0.20	0.05		0.19	0.61
weighted		0.32	0.07	0.07	0.21	0.18		0.58
pooling		0.68	0.57	0.57	0.62	0.61	0.57	

In this context, the Fisher method [47] was used to combine p-values in order to determine the overall significance of the differences between the distributions of the various methods. This method makes it possible to summarize the p-values from the Kolmogorov-Smirnov tests for the different risk scenarios (Table 3) and thus make an overarching statement about the significance of the differences.

**Table 3:** Combined p-Values using Fisher’s method for all risk scenarios.

aggregation method		classic	area	center	centertogrid	reachedgrid	weighted	pooling
		IMPACT						
classic	OCCURRENCE		0.00	0.00	0.00	0.00	0.00	0.00
area		0.00		0.07	0.00	0.00	0.97	0.00
center		0.00	1.00		0.00	0.00	1.00	0.00
centertogrid		0.00	0.00	0.00		0.99	0.00	0.00
reachedgrid		0.00	0.00	0.00	1.00		0.00	0.00
weighted		0.00	1.00	1.00	0.00	0.00		0.00
pooling		0.00	0.00	0.00	0.00	0.00	0.00	

Subsequently, the number of statistically significant method comparisons was determined for the risk scenarios and presented as a percentage in Table 4. In this study, a significance level of 0.05 was used to assess the statistical significance of the results.

**Table 4:** Percentage of non- significant results in the method comparison across all risk scenarios.

aggregation method		classic	area	center	centertogrid	reachedgrid	weighted	pooling
		IMPACT						
classic	OCCURRENCE		0.00	2.94	77.94	75.00	0.00	0.00
area		0.00		97.06	0.00	0.00	100.00	0.00
center		2.94	100.00		30.88	20.59	100.00	0.00
centertogrid		61.76	14.71	60.29		98.53	25.00	0.00
reachedgrid		42.65	0.00	48.53	100.00		10.29	0.00
weighted		2.94	100.00	100.00	50.00	29.41		0.00
pooling		0.00	0.00	0.00	0.00	0.00	0.00	

The Wilcoxon signed-rank test is used to statistically assess whether the central tendencies of the underlying populations are equal in two paired samples. In this specific case, the test examines whether there is a significant difference between the results of the method comparisons for individual scenarios, based on Kolmogorov-Smirnov distances for risks and opportunities. Table 5 presents the results of the individual method comparisons for both impact and probability of occurrence.

**Table 5:** p-Values of the method comparison between the risk and chance scenarios by Paired Wilcoxon Signed-Rank Test.

aggregation method		classic	area	center	centertogrid	reachedgrid	weighted	pooling
		IMPACT						
classic	OCCURRENCE		0.69	0.17	0.21	0.23	0.18	0.25
area		0.07		0.04	0.05	0.16	0.04	0.59
center		0.60	0.00		0.00	0.01	0.22	0.99
centertogrid		0.24	0.01	0.25		0.42	0.01	0.89
reachedgrid		0.10	0.80	0.11	0.00		0.00	0.06
weighted		0.40	0.00	0.00	0.11	0.71		0.63
pooling		0.18	0.10	0.38	0.28	0.33	0.41	

Despite the small differences in Kolmogorov distances of risks and opportunities, the statistical significance of the p-values is primarily due to the high number of cases, which results from the multiplication of the number of raters and the number of scenarios. This means that even minor differences can be classified as significant. Although there are some significant differences in the method comparison, these differences are very small and of limited relevance for practical purposes. The evaluation is based not only on statistical significance but also on the effect size. The effect size, in this case, represents the difference between the Kolmogorov distances for risks and opportunities, is crucial for determining whether a significant difference also has practical relevance. In our data, the maximum effect size was 0.03, while the average effect size was −0.011, indicating that the overall differences between the methods are minimal, concerning risk or opportunity assessment. Nevertheless, the differences between the aggregation methods, as the core focus of this paper, remain statistically significant.

## 9. Discussion

Our work addresses a need recently raised in [48], who called for future research on integrating uncertainty in visual tools, for the purpose of better risk communication; a problem that was independently discussed and confirmed in [49]. Beyond our medical context, the concept of measuring a “level of agreement” was also proposed in a recent study by [50]; the proposal of graphical risk assessment, studied in this work, brings these ideas closer to generic risk management standards, by generalizing the widely accepted methods proposed by [3,51,52], among others, to include uncertainty. However, with the demand for uncertainty modeling being eloquently discussed by [4], any generalization of well proven methods needs to make sure that it “covers” the original method as a special case (at least), i.e., does not deliver fundamentally different results. Our tests confirmed the method as one that ‘adds’ information for a decision-maker while ensuring that the risk assessment itself remains consistent with what would have been found by ‘classical’ means. Additionally, risk assessments conducted using graphical methods may help address the challenge of ‘verbalizing’ uncertainties. For example, the work of [53] studies membership functions for that purpose, which the graphical method naturally delivers. Most importantly, Dempster-Shafer theory has recently been identified as having high potential for risk management [54–56], but operates with intervals, beliefs, plausibility and possibility measures, all of which impose challenges for decision makers to specify. Such a graphical specification for generalized reasoning under uncertainty for critical infrastructures [57] must, as with any generalization, be consistent with what classical methods would deliver.

While our study analyzes statistical differences between methods, the absence of a definitive ground truth prevents a direct validation of the risk assessment results. Therefore, our focus was on assessing the consistency of individual subjective evaluations. This approach provides valuable insights into the reliability and reproducibility of different assessment methods, even in the absence of an absolute benchmark, which is crucial for improving risk assessment methodologies and enhancing their practical applicability.

Both the classical and graphical risk and opportunity assessment methods yield distinct results, although an examination of the results does not allow any statement to be made as to which assessment or aggregation method is the best. It is only possible to show the measurable differences on the basis of this survey and thus provide an overview of the different results. These measurable differences are reflected in aspects such as the precision or uncertainty of the evaluation, the comprehensibility of the results and the user-friendliness of the method.

In Table 2 comparing the Kolmogorov-Smirnov distances, the pooling method tends to show a higher distance compared to other methods. This indicates that the pooling method shows greater differences in the valuation results for both risk and opportunity scenarios. The weighted method often shows the lowest distances to other methods in both tables, particularly in relation to the area method, as it essentially derives from it. The Weighted method provides results that are very similar to those of the Area method. The Classical method shows moderate distances to other methods but remains relatively consistent in its placement. The variabilities of the Center-to-Grid and Reached-Grid methods show medium to high distances compared to other methods, indicating that they exhibit higher variation in their results. The choice of assessment and aggregation method should therefore depend on the specific requirements and objectives of the project in question. A combination of both approaches can sometimes be beneficial in obtaining a more balanced view of risk and opportunity assessment. A combination of different aggregation methods can also be useful. In summary, none of the methods can be considered superior per se. Rather, the specific framework conditions and requirements of the use case should be taken into account in order to select the most suitable method.
